# Variability of anti-staphylococcal antibodies in healthy volunteers and pre-cardiac surgery patients

**DOI:** 10.1186/s13741-016-0039-y

**Published:** 2016-05-27

**Authors:** Sarka Moravcova, Bonnie Kyle, Hilary Shanahan, Savvas Giannaris, Andrew Smith, Colin Hamilton-Davies

**Affiliations:** Royal Brompton & Harefield NHS Trust, London, UK; Barts Health NHS Trust, London, UK

**Keywords:** Staphylococcus, Antibody, Pre-operative, EndoCAb, Infection, Complication

## Abstract

**Background:**

Pre-operative antibody levels have been shown to be inversely related to development of post-operative complications. Staphylococcal infection is a major source of morbidity following surgery.

**Methods:**

We examined the variability of anti-staphylococcal antibody levels across a group of healthy volunteers and compared this with patients scheduled to undergo cardiac surgery.

**Results:**

Pre-operative cardiac surgical patients exhibited significantly higher levels of staphylococcal antibodies compared with healthy volunteers.

**Conclusions:**

The relationship between pre-surgery staphylococcal antibody levels and outcome warrants further investigation.

## Background

The incidence of wound infection following cardiac surgery is approximately 1–4 %, of which approximately one fifth is due to methicillin-resistant *Staphylococcus aureus* (MRSA) (Allen et al. 2014). Post-operative MRSA infection is associated with a mortality ranging from 10 to 14 % (Ridderstolpe et al. 2001). In addition, there is a considerable cost both economically and in terms of patient suffering that result from sternal wound breakdown, staphylococcal septicaemia and the associated increased length of hospital stay (Coskun et al. 2005). Kanafani demonstrated that whilst certain pre-operative characteristics were associated with an increased risk of post-operative staphylococcal sepsis, the majority of those that developed such complications had no such risk factors (Kanafani et al. 2009). These patients had no measure made of their underlying immunity to *Staphylococcus*. Similarly, in one of the largest retrospective studies of cardiac surgical patients (*n* > 330,000), there were again relationships drawn between pre-operative risk factors such as body mass index (BMI), diabetes, chronic renal insufficiency, increasing age and immunosuppressive therapy and development of infective complications (Fowler et al. 2005). In this study, again, no measure had been made of the patients’ own underlying ability to resist infection.

The humoral immune system provides a level of protection to patients against a number of infective/inflammatory conditions and indeed our own institution has been involved in demonstrating links between low levels of pre-operative antibody against endotoxin and outcome following cardiac surgery (Hamilton-Davies et al. 1997; Bennett-Guerrero et al. 1997). It is likely that everyone has a varying level of antibodies to common infective organisms, thus maintaining a degree of circulating immunity to these organisms, and that these antibodies are functional, i.e. have neutralising or opsonizing functions.

It has been shown that the alpha-toxin fragments secreted by *Staphylococcus* can impair gut mucosal integrity and thus further worsen the potential for sepsis by enabling Gram-negative organism/endotoxin translocation from the gut lumen (Kwak et al. 2012).

Anti-staphylococcal antibodies to a variety of epitopes can be measured by enzyme-linked immunosorbent assay (ELISA) (PhPlate AB, Stockholm, Sweden) (Colque-Navarro et al. 1998), and the levels of these antibodies are likely to vary between individuals. It is well established that there is considerable variation in antibody levels in healthy volunteers and in infected patients (Dryla et al. 2005). This is similar to the variation in levels of endogenous endotoxin antibodies, which are predictive of post-operative complications (Down et al. 2004).

We are particularly interested in those patients who develop serious *S. aureus* infections (deep-seated wound infection, serious bacteraemia, endocarditis) and examining whether there is a relationship with the patient’s own immune state. We set out to determine if these antibodies were measurable in individuals (healthy volunteers and pre-operative cardiac surgical patients) and whether there was measurable variation in antibody levels in these two groups. We planned to study the younger volunteers as they were likely to have an active, healthy immune system as compared to the patient group who may be expected to exhibit a degree of immunosenescence and have lower levels of circulating antibodies.

**Fig. 1:**
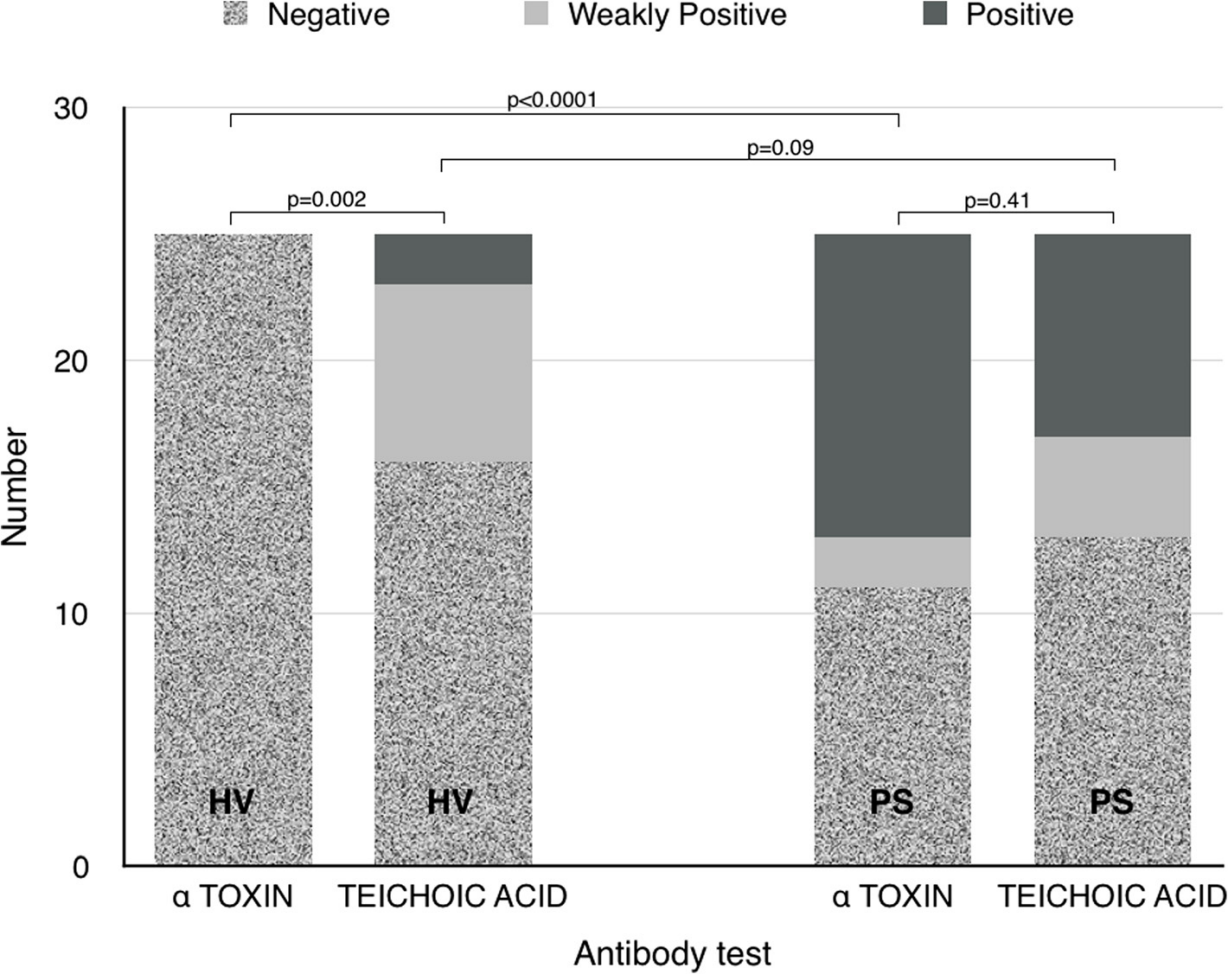
Categorised antibody response (positive, weakly positive, negative) to alpha-toxin and teichoic acid domains in healthy volunteers (HV), *n* = 25, and in pre-operative cardiac surgical patients (PS), *n* = 25. Fisher’s exact test (Freeman-Halton extension) used to test variability of response between groups

We chose to assay for antibodies to alpha-toxin (AT), an extracellular polypeptide, and to teichoic acid (TA), a major surface antigen of the staphylococcal organism; both are present in almost all strains of *S. aureus*. A high proportion (86–95 %) of the *S. aureus* isolates in clinical infections produce an anti-alpha-toxin antibody response (Mollby 1983) (Fig. 1). In cases of serious staphylococcal infection, the levels of alpha-toxin have been demonstrated to be very high, suggesting that the antigen is highly immunogenic (Soderquist BC-N et al. 1993). Teichoic acid is particularly expressed in case of long-standing staphylococcal infection, for example, deep-seated wound infection or endocarditis (Colque-Navarro et al. 1998). These two antibody types are likely to be reliably expressed in those patients that we are most interested in studying; those that develop deep-seated staphylococcal wound infections following cardiac surgery.

The IgG class of antibody is likely to be a more reliable indicator due to the reliability in appearance and longevity of response. Peak levels of the IgM class may be missed due to its more transient nature (Barclay 1995).

This investigation formed the first part of an ongoing investigation into peri-operative staphylococcal antibody levels and clinical outcome. As this was a pilot study and we were uncertain as to the degree of variation in antibody levels within and between the groups, we did not perform power calculations. The results of this investigation will help with the prediction of sample sizes in our future studies.

### Hypotheses

There is a measurable intra-population and inter-population variability in anti-staphylococcal antibodies amongst different populations of individuals.

## Methods

Ethical approval for this study was given by the Joint UCL/UCLH Committee on the Ethics of Human Research (Committee Alpha), REC reference 05/Q0502/72.

Prior to enrolment, verbal and written information was provided to all participants after which verbal and written consent to participate was obtained. No individual identifiable data is included in this submission.

Twenty-five healthy volunteers (HV) and 25 pre-operative cardiac surgery patients (PS) were recruited into the study, provided with study information and asked for written, informed consent for collection of 7 mL blood into a plain tube (Becton-Dickinson, Oxford, UK) for later analysis. Exclusion criteria were age under 18 years, recent or ongoing infection, pregnancy, an immunosuppressive condition and concomitant use of immunosuppressive therapy.

The patient group was consecutively recruited from a population scheduled to undergo a variety of cardiac surgical procedures. There were no refusals to participate in the study.

### Sample analysis

The 7-mL blood samples were allowed to clot at room temperature for 20 min, centrifuged for 10 min at 3000 rpm and the serum removed by pipette into cryotubes for storage at −70 °C for later analysis (Thermo Scientific, MA, USA). The ELISA procedure has been detailed previously. Briefly, coating doses for the 96-well microtitration plates (Dynatech M-129B, Plochingen, Germany) with α-toxin and teichoic acid were established at 2.5 and 1 μg/mL, respectively. The working volume throughout the tests was 100 μL/well. The microtitration plates were coated with antigens diluted in phosphate-buffered saline (PBS), pH 7.4, and incubated overnight at 22 °C. The plates were washed and a serum dilution in PBS with Tween-20 0.05 % *v/v* (PBS-T) of 1 in 1000 for α-toxin and a 1 in 10,000 for teichoic acid was added to the two coated wells. Positive and negative controls were included in each plate. The plates were incubated for 1 h at room temperature (20 °C). After washing the plates, alkaline phosphatase-conjugated goat anti-human antibody (Sigma) diluted in PBS-T was added to each well, and the plates were incubated for 2 h at room temperature. After the final wash, p-nitrophenyl-phosphate substrate (Sigma) was added. Titres were read when the positive controls reached previously established values at 405 nm on a Titertek Multiskan (Flow Laboratories, Irvine, Scotland) instrument. Antibody levels were categorised to a low, intermediate and high response to the teichoic acid and alpha-toxin domains. Statistical analysis comparing the variability in proportions of response between the groups was performed using the Freeman-Halton extension of Fisher’s exact test. Probabilities are two tailed.

## Results

A total of 25 healthy volunteers were recruited with a mean age of 41 years (range 27–51) and included 17 males and 8 females. Twenty-five pre-operative patients were also recruited with a mean age of 62 years (range 19–78) and included 17 males and 8 females. The surgical procedures undertaken on these patients were coronary artery bypass grafts (CABG)—9, valve replacement—10, CABG+ valve—5 and atrial septal defect repair—1.

Antibody levels to both AT and TA were obtained for all participants.

The patterns of distribution of each classification (low, intermediate or high) for AT and TA antibody levels appeared to differ between the two study groups (Fig. 1). A striking element of the results was the proportion of patients in the pre-surgery group with high antibody levels to alpha-toxin (48 % vs. 0) and to teichoic acid (32 vs. 8 %) when compared with the healthy volunteer group.

The distribution of classification for alpha-toxin in the HV group (low = 25, intermediate = 0, high = 0) differed significantly from that in the PS group (11, 2, 12, respectively); *p* < 0.0001. The distribution of each classification for TA antibody level in the HV group (16, 7, 2) did not vary significantly from that of the PS group (13, 4, 8); *p* = 0.09.

Amongst the HV group, the distribution of classification of response to AT (25, 0, 0) differed from that to TA (16, 7, 2); *p* = 0.002. However, in the PS group, the distribution of response to the two tests did not differ, AT (11, 2, 12) vs. TA (13, 4, 8); *p* = 0.41.

## Discussion

It is expected that antibody levels to an antigen will vary between individuals and that the response to various antigens will also vary in any given individual (Dryla et al. 2005). Both the healthy volunteers and the pre-operative patients were assumed to be in reasonable health with respect to any ongoing infection, and none were thought to have a concurrent infection at the time of study. The cohort of healthy volunteers had a significantly lower mean age than the pre-surgery group and would thus be reasonably expected to have higher antibody levels as, in general, downgraded immune responsiveness or immunosenescence is seen with advancing age (Haq and McElhaney 2014). This was not seen, and indeed, the pre-cardiac surgical group had a greater proportion of high antibody levels to both teichoic acid and alpha-toxin. Subjects with a recent infection may well have higher antibody levels than those in a non-infected group (Dryla et al. 2005). It may be that the pre-surgery group had been exposed to a significant staphylococcal challenge whereas the healthy cohort had not. Considering the different populations and looking at any obvious invasive procedure that the surgical group had undergone when compared to the healthy volunteers, we felt that cardiac catheterisation was the universally most invasive. It may be that the aseptic technique during the cardiac catheterization process, or during some other temporally related procedure such as peripheral venous cannulation, was inadequate. This hypothesis would need to be tested by comparing antibody levels before and after cardiac catheterisation procedures. There could, of course, be other factors that may lead to a differing level of antibodies between the groups. Pre-operative blood testing and venous cannulation will likely have occurred in the patient group.

It would also be interesting and useful to measure antibody levels in healthy volunteers of the same age range as pre-operative patients or perhaps measuring antibody levels in older patients not scheduled to undergo cardiac surgery. Any observed differences between these two groups may imply that another mechanism might be the cause of antibody level variation.

The variation in antibody levels in the pre-operative patient group, all of whom had a similar potential inoculative insult, may be due to a natural variation in antibody levels between individuals but also may represent variability in the individual’s ability to generate an immune response. Some patients generated a high level of antibody and others generated low or no response. Do those patients with low antibody levels suffer a different level of peri-operative complication than those with higher levels?

The association between low pre-operative antibody levels and compromised outcome following cardiac surgery has already been made for endotoxin core antibodies (EndoCAb) (Hamilton-Davies et al. 1997; Bennett-Guerrero et al. 1997). In this pilot study, no outcome comparisons could be made due to small numbers and heterogeneity of both the patients and the surgical procedures. The variability in underlying immunity to both teichoic acid and alpha-toxin observed in pre-cardiac surgery patients suggests that a controlled outcome study be performed in this group. The ability of pre-operative staphylococcal antibody levels to predict post-operative infectious outcomes could be enhanced by perhaps combining with other antibody levels (e.g. EndoCAb).

If an inverse relationship between pre-operative anti-staphylococcal antibody levels and subsequent development of post-cardiac surgery complications exists, similar to that for EndoCAb, it may be possible to intervene to modify the post-procedural outcome.

It has been previously reported by Colque-Navarro and colleagues that those patients with low levels of antibodies, especially to alpha-toxin, may not mount much of a response to antigenic exposure (Colque-Navarro et al. 1998). This may reflect a more generalised immune hypo-responsiveness where active vaccination might not generate an adequate response. Passive immune therapy, such as the administration of a gammaglobulin product, may be more appropriate. It may be that for patients with a deficient pre-operative immune status, an alternative, less invasive, surgical or non-surgical intervention may be available and appropriate.

## Conclusions

This demonstration of the variability in anti-staphylococcal antibody levels suggests that the relationship between pre-surgery staphylococcal antibody levels and outcome warrants further investigation. Predicting which patients are more likely to suffer a complicated course following surgery might enable either more effective redirection of those patients to other appropriate treatment modalities or targeting specific resources to those patients to give them the best chance of dealing with the surgical insult.

## Abbreviations

AT: alpha-toxin
BMI: body mass index
CABG: coronary artery bypass grafts
ELISA: enzyme-linked immunosorbent assay
HV: healthy volunteers
MRSA: methicillin-resistant *Staphylococcus aureus*
PBS: phosphate-buffered saline
PS: pre-operative cardiac surgery patients
TA: teichoic acid
